# Characterization of GSK′963: a structurally distinct, potent and selective inhibitor of RIP1 kinase

**DOI:** 10.1038/cddiscovery.2015.9

**Published:** 2015-07-27

**Authors:** SB Berger, P Harris, R Nagilla, V Kasparcova, S Hoffman, B Swift, L Dare, M Schaeffer, C Capriotti, M Ouellette, BW King, D Wisnoski, J Cox, M Reilly, RW Marquis, J Bertin, PJ Gough

**Affiliations:** 1 Pattern Recognition Receptor Discovery Performance Unit, Immuno-inflammation Therapeutic Area, GlaxoSmithKline, Collegeville, PA, USA; 2 Platform Technology and Science, GlaxoSmithKline, Collegeville, PA, USA

## Abstract

Necroptosis and signaling regulated by RIP1 kinase activity is emerging as a key driver of inflammation in a variety of disease settings. A significant amount has been learned about how RIP1 regulates necrotic cell death through the use of the RIP1 kinase inhibitor Necrostatin-1 (Nec-1). Nec-1 has been a transformational tool for exploring the function of RIP1 kinase activity; however, its utility is somewhat limited by moderate potency, off-target activity against indoleamine-2,3-dioxygenase (IDO), and poor pharmacokinetic properties. These limitations of Nec-1 have driven an effort to identify next-generation tools to study RIP1 function, and have led to the identification of 7-Cl-O-Nec-1 (Nec-1s), which has improved pharmacokinetic properties and lacks IDO inhibitory activity. Here we describe the characterization of GSK′963, a chiral small-molecule inhibitor of RIP1 kinase that is chemically distinct from both Nec-1 and Nec-1s. GSK′963 is significantly more potent than Nec-1 in both biochemical and cellular assays, inhibiting RIP1-dependent cell death with an IC_50_ of between 1 and 4 nM in human and murine cells. GSK′963 is >10 000-fold selective for RIP1 over 339 other kinases, lacks measurable activity against IDO and has an inactive enantiomer, GSK′962, which can be used to confirm on-target effects. The increased *in vitro* potency of GSK′963 also translates *in vivo*, where GSK′963 provides much greater protection from hypothermia at matched doses to Nec-1, in a model of TNF-induced sterile shock. Together, we believe GSK′963 represents a next-generation tool for examining the function of RIP1 *in vitro* and *in vivo*, and should help to clarify our current understanding of the role of RIP1 in contributing to disease pathogenesis.

RIP1 has a role as an essential adaptor for signaling induced by a number of innate immune receptors including TNFR1^1^ and other death receptors, TLR3^2^ and TLR4.^3^ Studying the role of RIP1 in these pathways has revealed very distinct functions for RIP1 as a scaffolding protein^4^ and as a kinase.^5–7^ Although the scaffolding function of RIP1 is essential for restraining the activation of cell death pathways under homeostatic conditions,^8,9^ the kinase activity of RIP1 is emerging as a driver of inflammation and tissue damage in a variety of disease settings, most notably involving the proinflammatory cytokine TNF.^10,11^ The pathogenic effects of RIP1 kinase activity have been attributed to its role in regulating an inflammatory form of necrotic cell death termed necroptosis.^12^ However, data are emerging to suggest that RIP1 kinase activity can also regulate apoptosis^13^ and the production of inflammatory cytokines^14^ under certain circumstances, and these pathways may also contribute to the role of RIP1 kinase activity in driving inflammation.

Interest in the role that RIP1 kinase activity has in driving disease pathogenesis was sparked by the identification of the small-molecule RIP1 kinase inhibitor Necrostatin-1 (Nec-1) from a phenotypic screen looking for inhibitors of necroptosis.^12,15^ Other small-molecule inhibitors of RIP1 kinase have since been identified, but lack the kinase selectivity observed with Nec-1.^16–18^ Nec-1 is a small, tryptophan-based compound that demonstrates remarkable selectivity for RIP1 over other kinases. It has been used extensively by many groups to elucidate the role of RIP1 kinase activity and necroptosis both in *in vitro* and *in vivo* assays.^19–21^ Despite its broad use, Nec-1 is not an ideal tool for studying RIP1 kinase function, as it has a number of limitations that may confound some of the original findings using the inhibitor. Nec-1 is only modestly potent, and often used at concentrations *in vitro*, which can lead to off-target effects. To this end, Nec-1 is known to inhibit indoleamine-2,3-dioxygenase (IDO), a molecule with important immunomodulatory activities.^22^ Furthermore, the metabolic stability of Nec-1 is quite limited, resulting in a short *in vivo* half-life.^23^ Together these suboptimal properties of Nec-1 have led to an effort to identify next-generation inhibitors of RIP1 kinase to provide superior *in vitro* and *in vivo* tool molecules. These efforts have led to the identification of 7-Cl-O-Nec-1 (Nec-1s), which lacks IDO activity and has improved pharmacokinetic properties.^24^ However, Nec-1s is still only modestly potent and is structurally related to Nec-1 and therefore may suffer from similar off-target activities.

In the present study, we characterize GSK′963, a novel small-molecule inhibitor of RIP1 kinase that is structurally distinct from Nec-1 and Nec-1s. GSK′963 is over 200-fold more potent than Nec-1, displays exquisite selectivity for RIP1 kinase activity and has no effect on IDO activity or on TNF-mediated NF*κ*B activation or apoptosis. GSK′963 is a chiral molecule allowing for use of its chemically identical inactive enantiomer as a negative control to confirm on-target activity of the inhibitor. Furthermore, GSK′963 provides complete protection in an *in vivo* model of TNF-induced sterile shock at a dose where Nec-1 shows no significant protection in the model. Taken together, we believe that GSK′963 represents an additional next-generation tool for exploring the role of RIP1 kinase-mediated biology.

## Results

### GSK′963 is a potent and selective inhibitor of RIP1 kinase in biochemical assays

**Figure 1:**
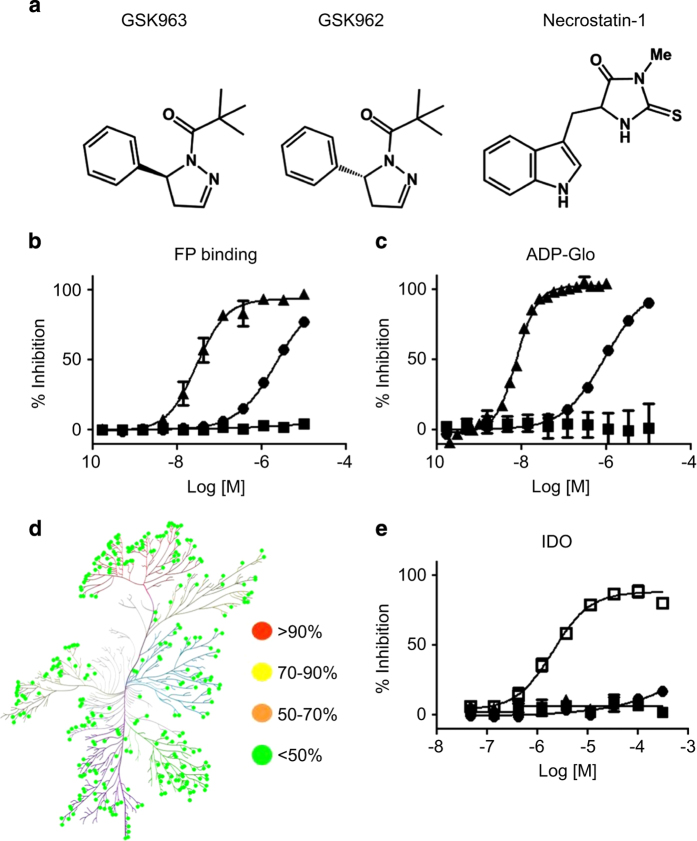
GSK′963A is a potent and selective inhibitor of RIP1 kinase. (**a**) Chemical structures of GSK′963A (active analog), GSK′962A (inactive analog) and Necrostatin-1. (**b**) Dose–response curves for GSK′963, GSK′962 and Nec-1 in the FP binding assay evaluating the affinity of compounds for RIP1 (ATP-binding pocket). Graphs represents *n*=52 for Nec-1, *n*=4 for GSK′962A and *n*=7 for GSK′963A. Error bars indicate S.D. (**c**) Dose–response curves for GSK′963, GSK′962 and Nec-1 in ADP-Glo kinase assay measuring autophosphorylation of RIP1 kinase domain *in vitro*. Graph represents *n*=20 for Nec-1, *n*=2 for GSK′962A and *n*=2 for GSK′963A. Error bars indicate S.D. (**d**) GSK′963 does not block the activity of any of the tested 339 human kinases at 10*μ*M concentration, as evaluated at the Reaction Biology Corporation. Each dot represents an individual kinase with the color indicating the level of inhibition (red>90%, yellow 70–90%, orange 50–70% and green <50%) (**e**) Effect of GSK′963, GSK′962 and Nec-1 on indoleamine 2,3-dioxygenase (IDO) activity evaluated by an *in vitro* enzymatic assay. Menadione was used as a positive control for IDO inhibition. The results are representative of three independent experiments. 
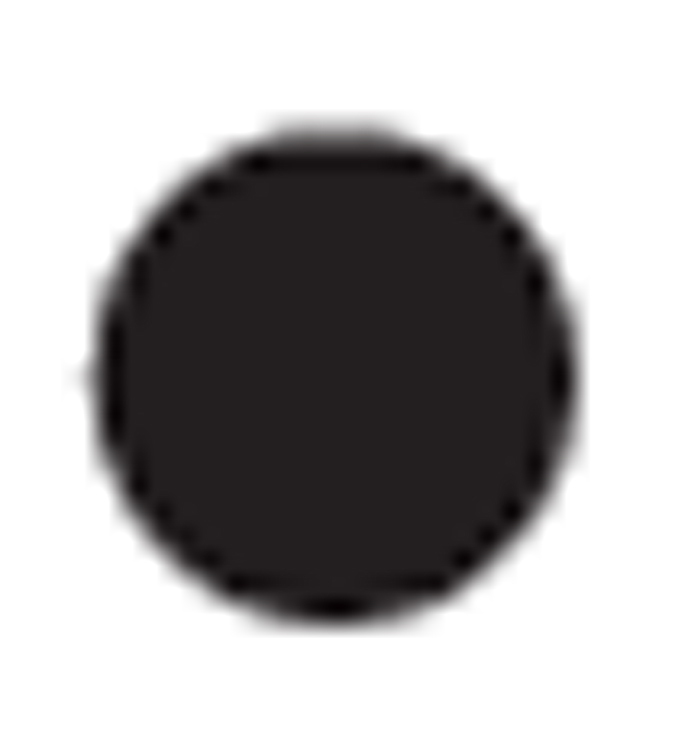
=Nec-1, 
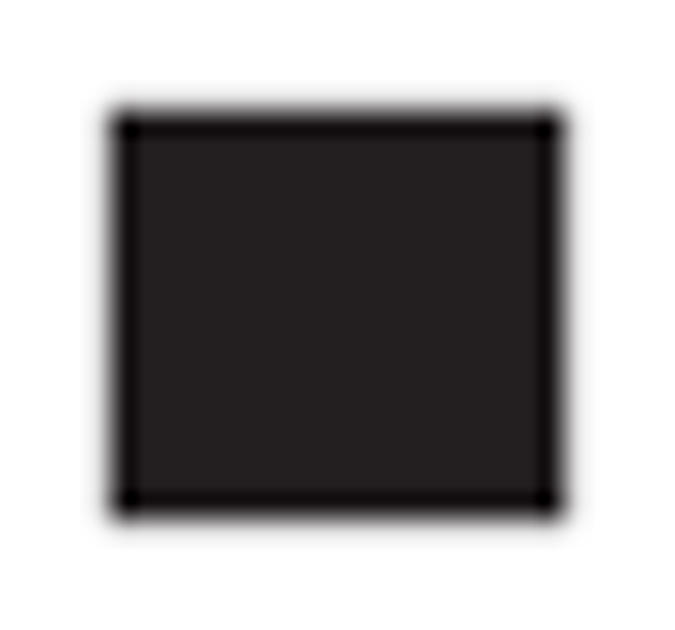
=GSK′962, 
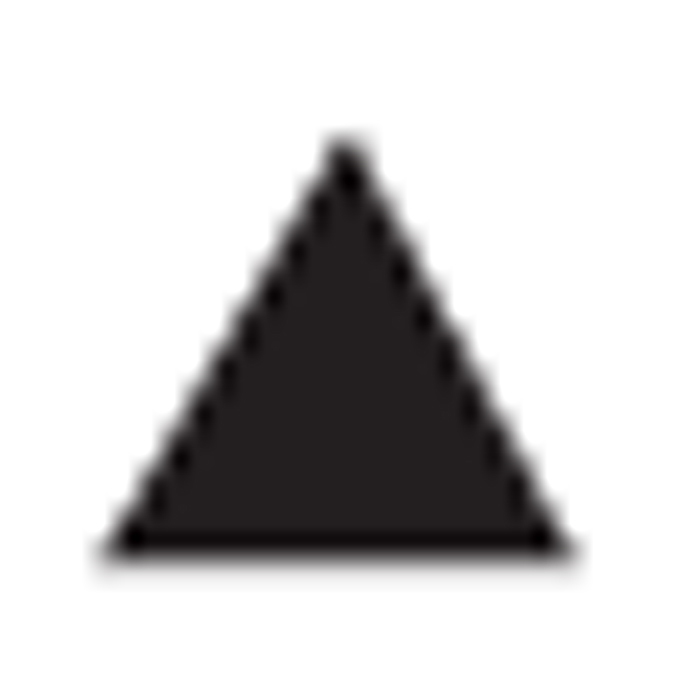
=GSK′963 and 
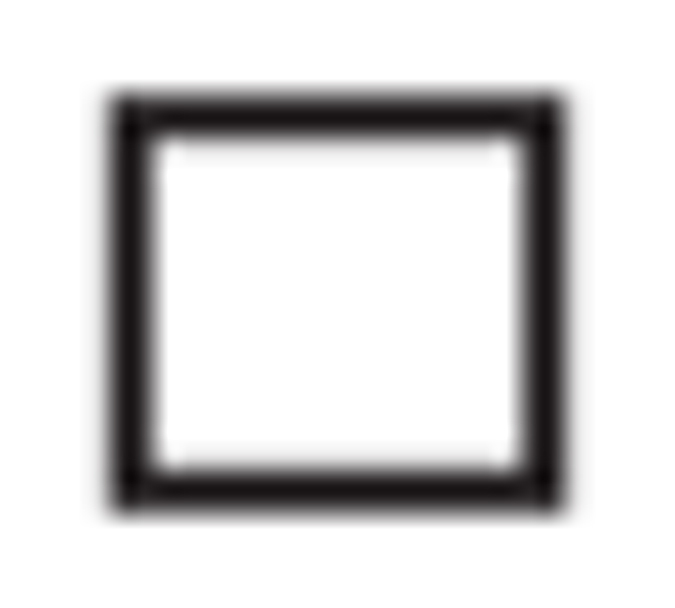
=Menadione.

To identify novel inhibitors of RIP1 kinase, we screened the GSK compound collection, containing ~2 million compounds, for RIP1 inhibitors using an ADP-Glo enzymatic assay. Minor modification of a screening hit led to the identification of a racemic compound that was subsequently separated into active (GSK′963) and inactive (GSK′962) enantiomers (Figure 1a). GSK′963 was shown to be highly potent in the FP binding assay (IC_50_=29 nM), above the limit of detection for this assay. This contrasted markedly to Nec-1 and GSK′962 (Figure 1a), which displayed an ~70-fold drop off in potency (IC_50_=2 *μ*M) or no activity in the assay, respectively, (Figure 1b). In accordance with the binding data, GSK′963 was also highly potent (IC_50_=8 nM with a 4 parameter curve fit or 0.8 nM with a tight binding fit) in blocking RIP1 kinase activity in comparison with Nec-1 (IC_50_=1 *μ*M) and GSK′962, which was inactive (Figure 1c).

Generating selective inhibitors of kinases has historically been challenging and therefore, we assessed the selectivity of GSK′963 against a panel of 339 kinases in activity assays at 10 *μ*M. GSK′963 was determined to be an ultra-selective RIP1 inhibitor, displaying <50% inhibition against all other kinases tested (Figure 1d; Supplementary Table I). Furthermore, GSK′963 was also inactive against indoleamine-2,3-dioxygenase (IDO) activity. This is in contrast to Nec-1, which has been reported to be a potent inhibitor of IDO.^25^ However, in our assays, Nec-1 also had no effect on IDO activity, whereas menadione, a natural inhibitor of IDO, inhibited enzyme activity with the expected potency (Figure 1e). Together these data demonstrate that GSK′963 is a highly selective tool molecule with greater potency than Nec-1 in biochemical assays.

### GSK′963 is a selective and potent inhibitor of necroptosis in murine and human cells *in vitro*

**Figure 2:**
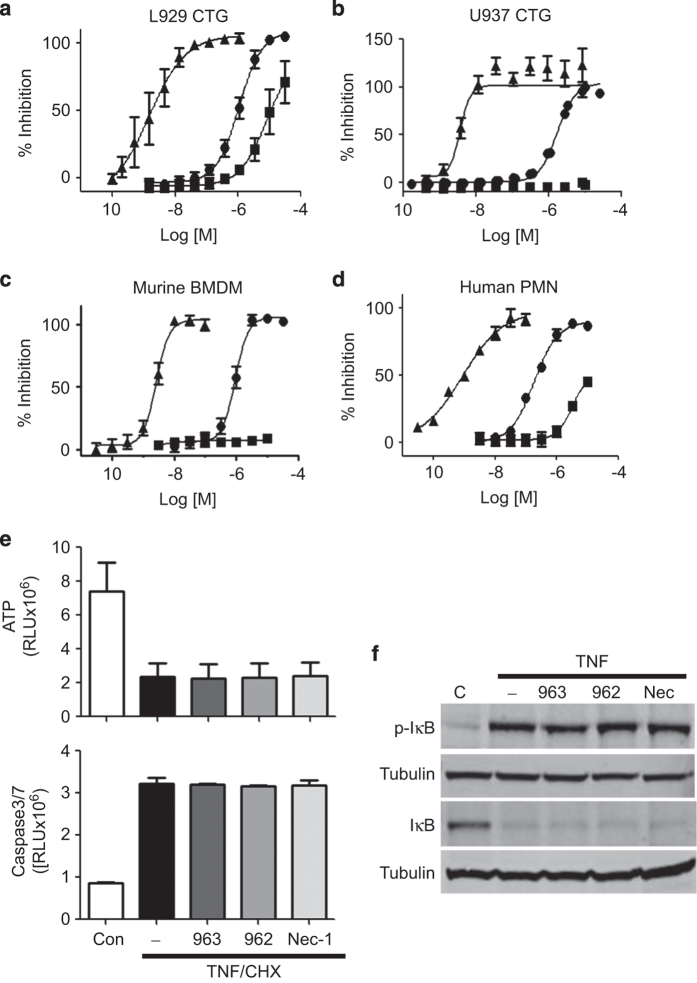
GSK′963A is highly potent in human and mouse cell-based assays and selective for inhibition of necroptosis. (**a–d**) Dose–response curves for GSK′963, GSK′962 and Nec-1 in cell-based assays. Necroptosis induced with TNF and zVAD in (**a**) mouse fibrosarcoma L929 cells, (**b**) human monocytic U937 cells and (**c**) primary murine bone marrow-derived macrophages was evaluated by measuring cell viability using CellTiter-Glo assay. (**d**) Primary human neutrophils were stimulated with TNF, zVAD and SMAC mimetic to induce necroptosis. Cell viability was evaluated as in **a**. The graphs represent combined data from at least three independent experiments. Error bars represent S.D. (**e**) Cell viability and Caspase 3/7 activity were measured in BMDM treated with TNF and cycloheximide. Cell viability was measured using the CellTiter-Glo assay at 20 h, Caspase 3/7 activity using the Caspase-Glo 3/7 assay at 3 h. GSK′963 and GSK′962 were used at 100 nM and Nec-1 was used at 10 *μ*M. Similar data were generated in four independent experiments. Error bars represent S.D. between two experiments measured on the same plate. (**f**) Western blot analysis of I*κ*B phosphorylation and degradation in BMDM stimulated with TNF. I*κ*B phosphorylation was evaluated at 5 min and I*κ*B degradation at 15 min. Tubulin was used as a loading control. GSK′963 and GSK′962 were used at 100 nM and Nec-1 was used at 10 *μ*M. Data are representative of experiments from four different animals. 
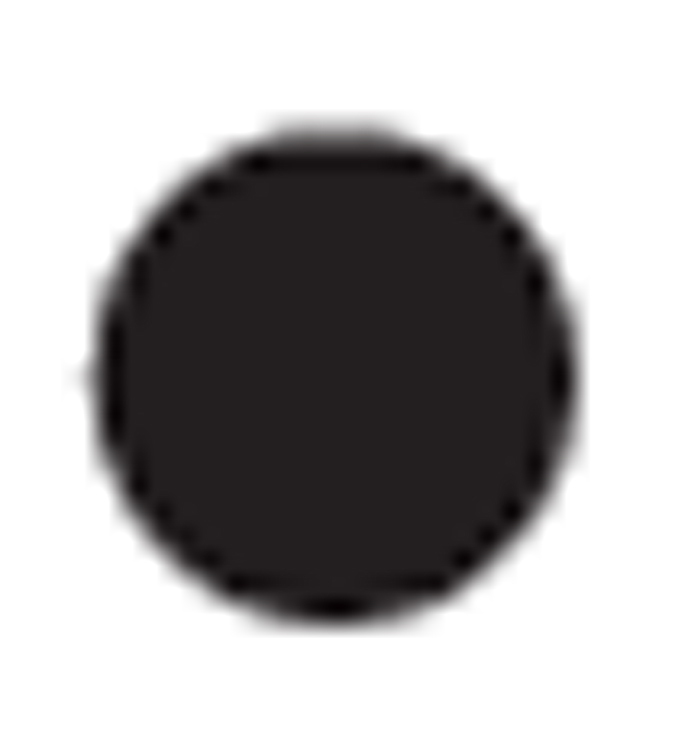
=Nec-1, 
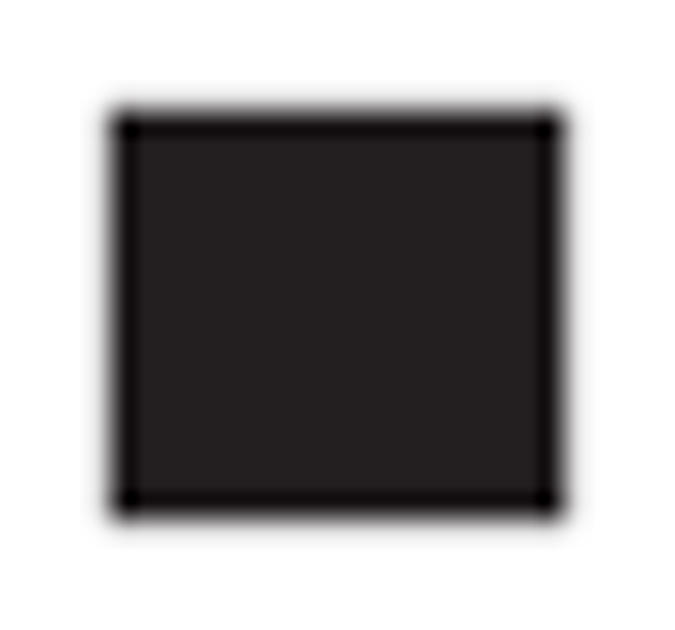
=GSK′962, and 
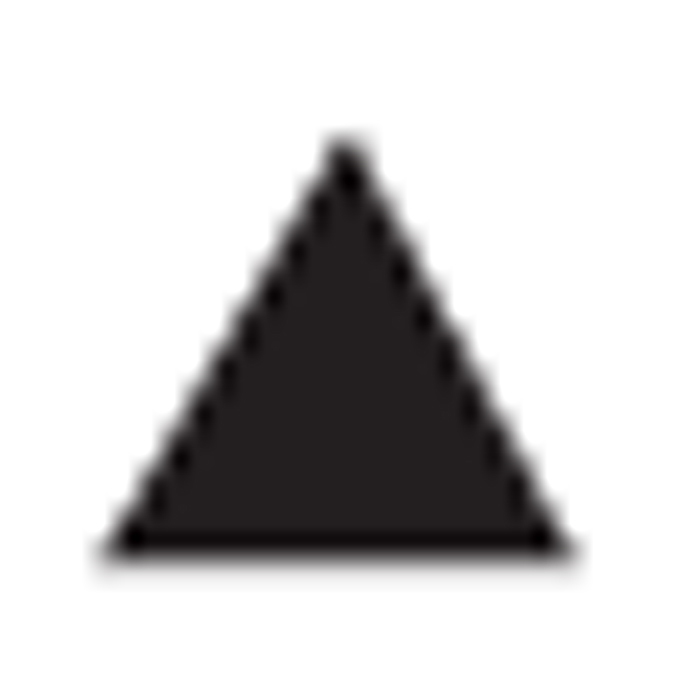
=GSK′963. C, control; CHX, cycloheximide.

We next assessed the ability of GSK′963, GSK′962 and Nec-1 to block necroptosis in murine and human cell lines *in vitro*. To this end, mouse L929 and human U937 cells were stimulated with TNF+zVAD to induce necroptosis, and the ability of compounds to block cell death was evaluated overnight. GSK′963 efficiently blocked necroptosis in both murine and human cells with IC_50_ values of 1 nM and 4 nM, respectively, whereas the inactive analog GSK′962 was at least 1000-fold less potent in these assays (Figures 2a and b). Nec-1 was significantly less potent as compared with GSK′963, displaying IC_50_ values of 1 *μ*M and 2 *μ*M in L929 and U937 cells, respectively. The potency of the compounds was confirmed in primary mouse bone marrow-derived macrophages (BMDMs) and primary human neutrophils stimulated to induce necroptosis. In good agreement with the cell line data, the IC_50_ values were determined to be 3 nM for the primary mouse BMDMs and 0.9 nM for primary human neutrophils, with Nec-1 and GSK′963 being significantly less potent or inactive in the assays (Figures 2c and d).

Previous data have shown that TNF-induced activation of NF-*κ*B, and TNF+cycloheximide (CHX)-stimulated apoptosis, occur independently of RIP1 kinase activity.^5^ We therefore used these assays to examine the cellular selectivity of GSK′963. Treatment with GSK′963 at 100 nM showed no measurable effects on TNF+CHX-stimulated apoptosis or TNF-induced NF*κ*B activation (Figures 2e and f). These results in conjunction with the wider kinase profiling data (Figure 1d; Supplementary Table I) demonstrate that GSK′963 is a highly potent and selective inhibitor of RIP1 kinase activity.

### GSK′963 is a potent inhibitor of a TNF+zVAD-mediated lethal shock

**Figure 3:**
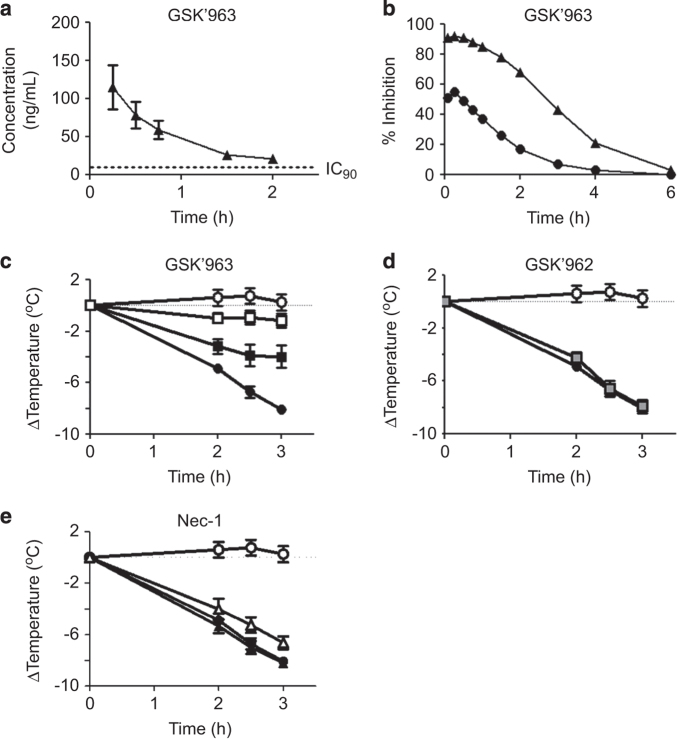
GSK′963A protects mice from TNF+zVAD-induced hypothermia. (**a**) Pharmacokinetic profile of GSK′963A dosed i.p. at 10 mg/kg in C57BL/6 mice. The data represent the combined results of the three independent animals. (**b**) Modeling of predicted % inhibition against RIP1 using the observed pharmacokinetic profile of GSK′963 in conjunction with the potency in inhibiting TNF+zVAD necroptosis in mouse L929 cells. 
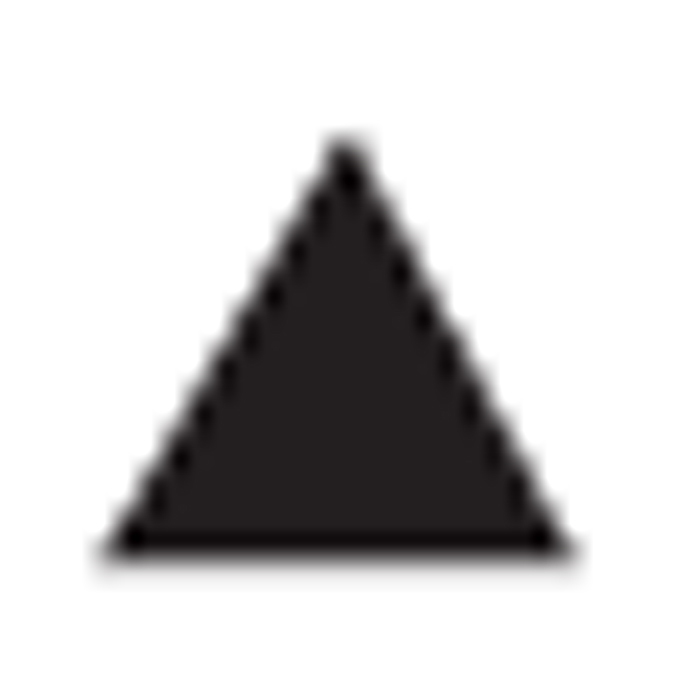
=2 mg/kg and 
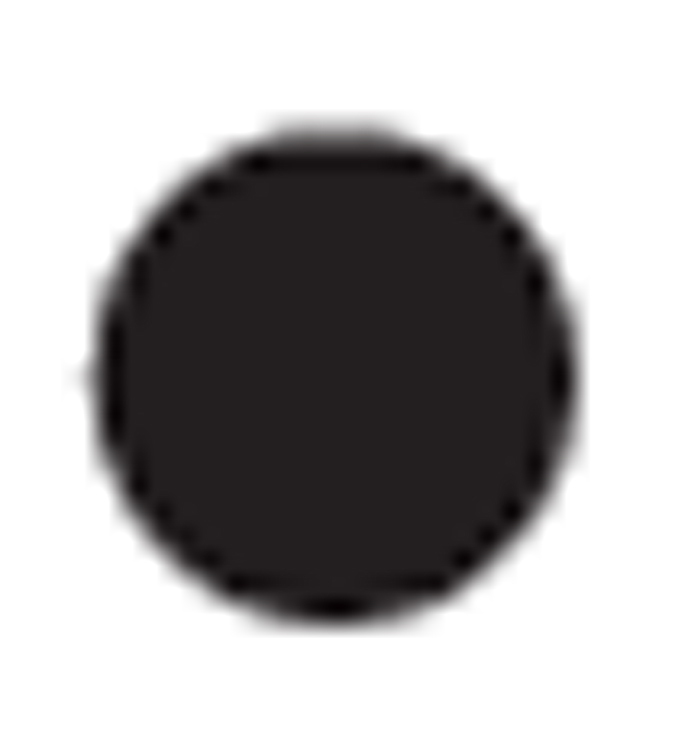
=0.2 mg/kg (**c–e**) The effect of GSK′963, GSK′962 and Nec-1 on temperature loss in the TNF+zVAD-induced shock model. C57BL/6 mice were pretreated i.p. with (**c**) GSK′963, (**d**) GSK′962A or (**e**) Nec-1 15 min prior to i.v. injection of TNF+zVAD. Temperature was monitored over the course of 3 h by rectal probe. The results are representative of three independent experiments, each containing seven animals per group. Error bars indicate the S.D. from the seven animals for each group. 
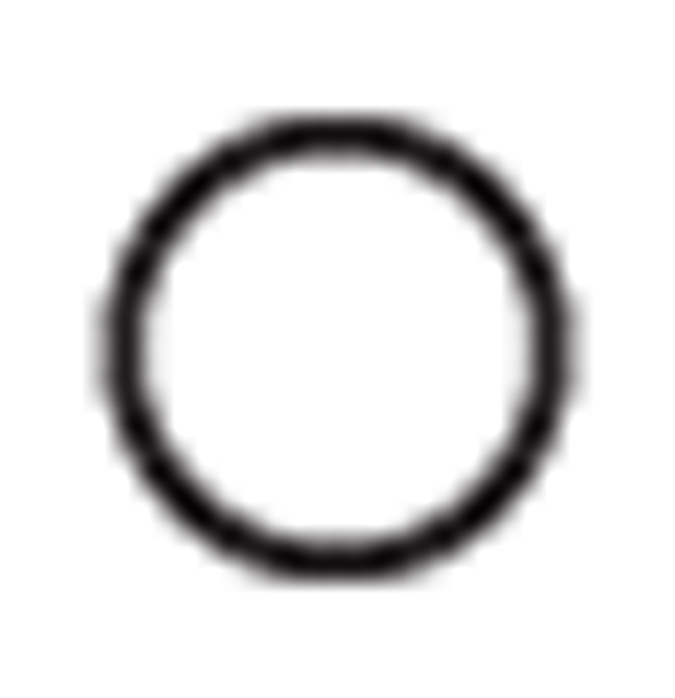
=control mice, 
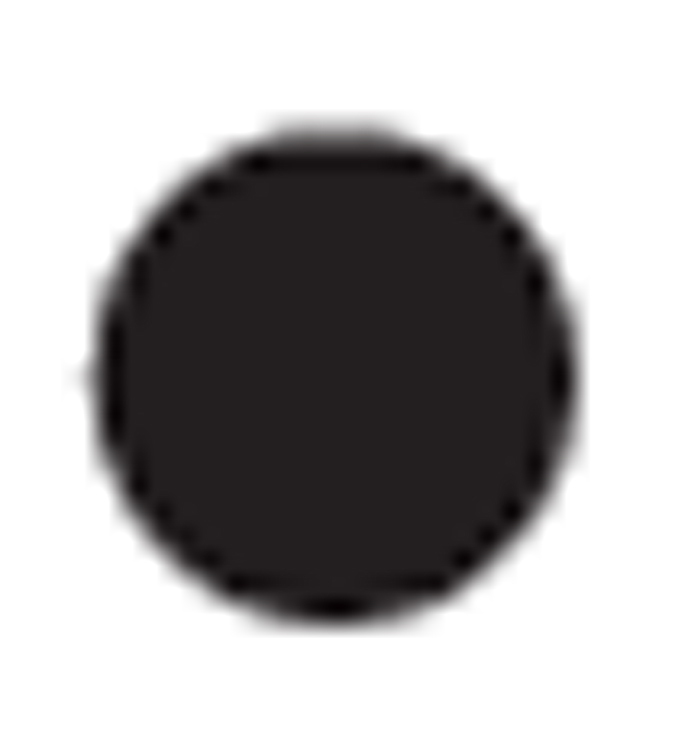
=TNF+zVAD-treated mice, 
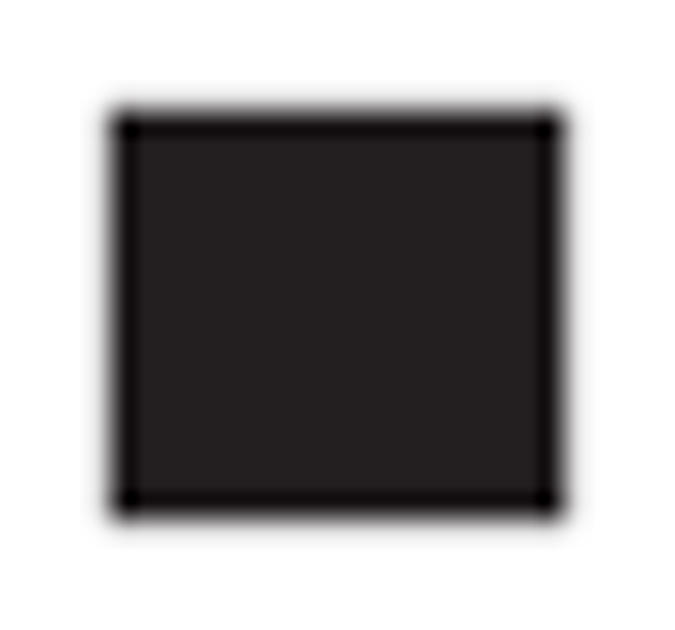
=TNF+zVAD-treated mice dosed with GSK′963 at 0.2 mg/kg, 
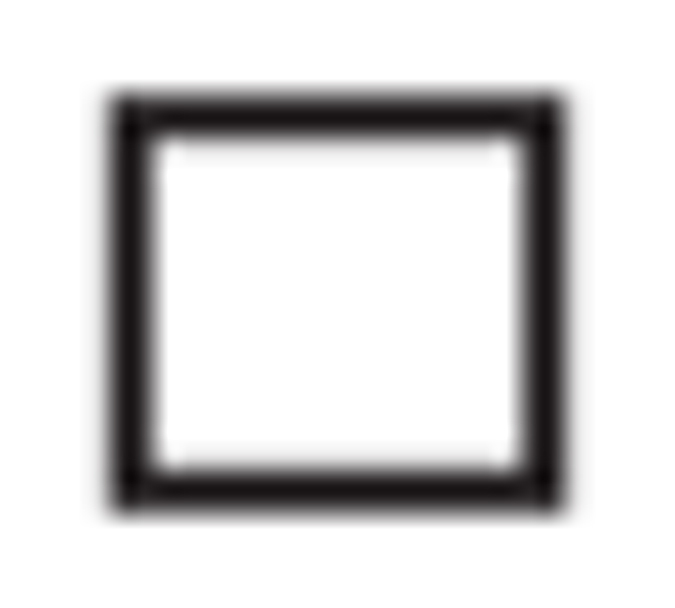
=TNF+zVAD-treated mice dosed with GSK′963 at 2 mg/kg, 
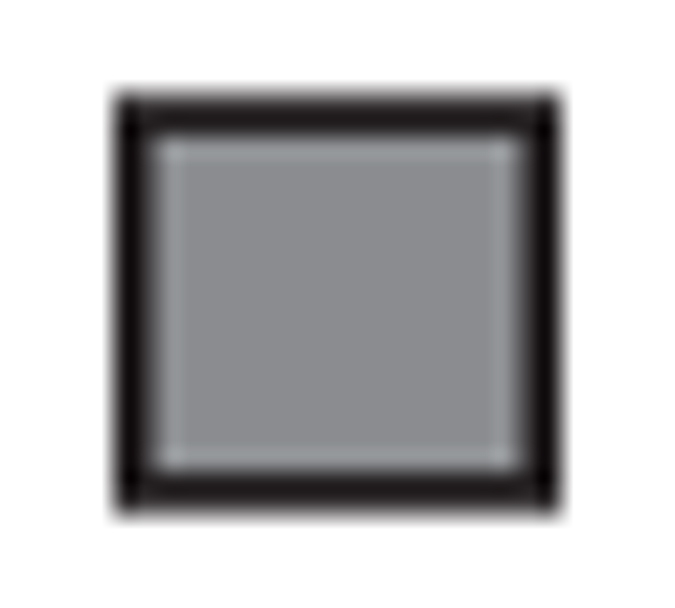
=TNF+zVAD-treated mice dosed with GSK′962 at 20 mg/kg, 
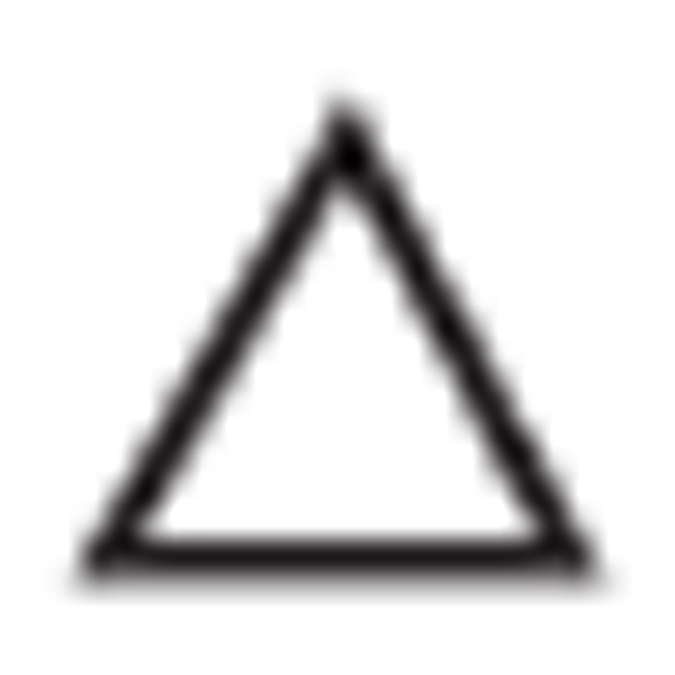
=TNF+zVAD with Nec-1 at 2 mg/kg and 
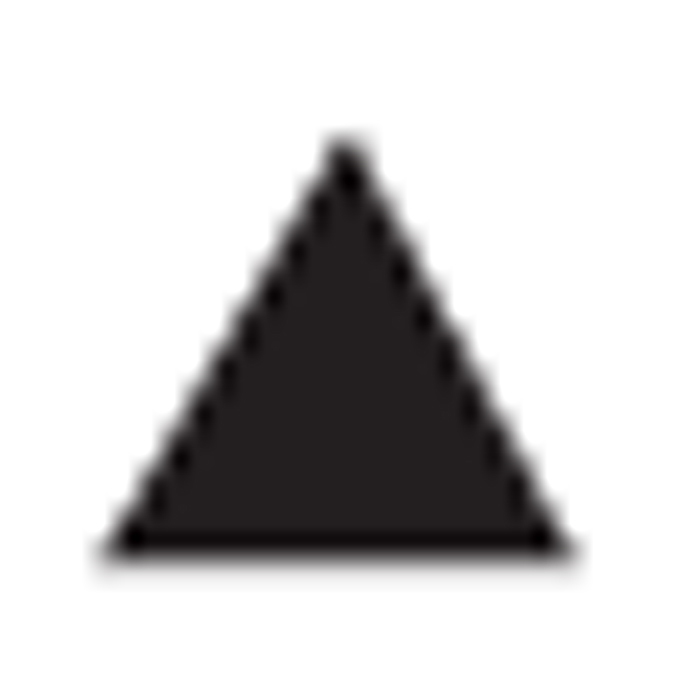
=TNF+zVAD with Nec-1 at 0.2 mg/kg.

We next assessed the pharmacokinetic profiles of GSK′963 and Nec-1 following intraperitoneal (i.p.) administration *in vivo*. Nec-1 demonstrated an ~10-fold higher exposure compared with GSK′963 at 10 mg/kg, although the apparent half-life of GSK′963 was greater than that for Nec-1 (Figure 3a; Supplementary Figure 1a). However, pharmacodynamic modeling of both compounds based on the mouse pharmacokinetic profiles (Figure 3a; Supplementary Figure 1a) and compound potencies in TNF+zVAD-treated L929 cells (Figure 2a) indicated that at 2 mg/kg, GSK′963 would maintain blood concentrations above the concentration required for 90% inhibition of RIP1 activity for an extended period of time compared with Nec-1 (Figure 3b; Supplementary Figure 1b). To directly test the efficacy of GSK′963 *in vivo*, we made use an acute model of sterile shock. Administration of TNF+zVAD results in a lethal hypothermia that has previously been shown to be dependent on RIP1 kinase activity.^5,18^ Treatment of animals with 2 mg/kg of GSK′963 resulted in a complete protection from TNF+zVAD-induced temperature loss, with the 0.2 mg/kg dose also showing a significant response (Figure 3c). As expected, GSK′962 had no effect on the TNF+zVAD-induced shock, confirming that GSK′963 was acting selectively through RIP1 kinase inhibition (Figure 3d). Interestingly, Nec-1 had no effect in the model at 0.2 mg/kg, a dose that is commonly used to inhibit RIP1 *in vivo* in the literature, and showed a minimal level of protection at a 10-fold higher dose (Figure 3e). Together these results demonstrate that compared with Nec-1, GSK′963 represents a better tool molecule to explore acute RIP1 kinase biology *in vivo*.

## Discussion

Over the last few years, RIP1 kinase-dependent necroptosis and signaling has emerged as a key driver of inflammation and disease pathogenesis.^10,11^ In parallel with the data supporting an inflammatory role for RIP1 kinase activity, the complexities surrounding RIP1-dependent signaling pathways have become better appreciated. It is now clear that RIP1 has very distinct roles as a scaffolding protein and as a kinase, with this most readily seen in the very distinct phenotypes of RIP1-deficient and RIP1 kinase-dead knock-in mice.^5–7,26^ In addition, it was initially suggested that the sole role of RIP1 kinase activity was to regulate RIP3-dependent necroptosis, and hence RIP3-deficient mice were assumed to be a surrogate of the absence of RIP1 kinase activity.^27^ However, recent data suggest that RIP3-deficient and RIP1 kinase-dead knock-in mice can have different phenotypes in inflammatory models, and that RIP1 kinase activity can also be involved in regulating apoptosis and the production of inflammatory mediators.^6,13,14^ Although these genetic systems are powerful, small-molecule inhibitors are extremely important in understanding the biology of RIP1 in situations where genetic mutants cannot be used, particularly in human cell systems.

The RIP1 inhibitor Nec-1 has been widely used in *in vitro* cellular systems, and has led to many important insights into RIP1 function.^15^ However, the moderate potency of Nec-1 has often led to it being utilized at concentrations of tens of micromolar. At these concentrations, it becomes hard to confidently discern whether the biological effects of Nec-1 are being mediated through inhibition of RIP1, or through off-target activities such as inhibition of IDO. Although optimized necrostatins, such as Nec-1s, have been generated, which are devoid of IDO activity, their moderate potency still raises questions about on-target *versus* off-target activity.^25^ In this regard, we believe that GSK′963 represents a significant advance for studying RIP1 function in *in vitro* settings, due to its low nanomolar activity in human- and murine cell-based assays, its exquisite kinase activity, and the ability to use the inactive enantiomer GSK′962 to control for off-target activities.^28^

The increased potency of GSK′963 relative to Nec-1 *in vitro* also translated into increased efficacy in the TNF+zVAD-induced model of sterile shock. At a dose of 2 mg/kg, GSK′963 was able to completely inhibit temperature loss in response to TNF+zVAD, recapitulating the phenotype of the recently described RIP1 kinase-dead knock-in mice. The level of inhibition of temperature loss in this acute model at different doses of GSK′963 was in line with the predictions of efficacy based on modeling of the pharamacokinetic profile and *in vitro* potency of GSK′963 (Figures 3b and c). Extending this modeling out over 24 h revealed that multiple, large doses of GSK′963 would be required to provide significant levels of RIP1 inhibition, making it unsuitable as a tool to study RIP1 function in chronic models of inflammation and disease (data not shown). We are currently working to identify analogs of GSK′963 with improved metabolic stability and pharmacokinetic properties that may represent superior tools for studying RIP1 function *in vivo*.

In our hands, administration of Nec-1 at doses commonly used in the literature to study RIP1 function had minimal effect in the RIP1-dependent TNF+zVAD model of sterile shock. This lack of efficacy was in agreement with the modeled RIP1 inhibition for Nec-1 (Supplementary Figure 1b) and reflects the moderate potency, and metabolic instability of Nec-1 and the corresponding short *in vivo* half-life for the compound. Although it is possible that modifications to the vehicle used to dose Nec-1 may modestly increase the levels of compound around the time of dosing, administration of Nec-1 at doses used in the literature will only be able to generate moderate, short-lived inhibition of RIP1 in chronic *in vivo* models. In this regard, many of the models of inflammation and disease, where RIP1 inhibition by Nec-1 has shown benefit, should be reevaluated when further optimized RIP1 inhibitors are available, in order to define the effects of more complete inhibition of RIP1 kinase activity. Furthermore, it will be informative for the field if future publications of *in vivo* studies using Nec-1 contain measurements of circulating compound levels in order to better understand the magnitude of RIP1 inhibition achieved.

Taken together, we have identified a novel, highly potent and selective inhibitor of RIP1 kinase activity. We believe that GSK′963 represents a next-generation tool inhibitor for investigating RIP1 biology *in vitro*, offering a significant benefit over the commercially available necrostatins.

## Materials and Methods

### Mice

C57BL/6 mice were purchased from Taconic (Hudson, NY, USA) and housed under specific pathogen-free conditions. All animal procedures were conducted in an Association for Assessment and Accreditation of Laboratory Animal Care-accredited facility at GlaxoSmithKline in accordance with the GlaxoSmithKline Policy on the Care, Welfare, and Treatment of Laboratory Animals and were reviewed and approved by the Institutional Animal Care and Use Committee at GlaxoSmithKline.

### Fluorescent polarization (FP) binding assay

A FP-based binding assay was developed to quantitate interaction of novel test compounds at the ATP-binding pocket of RIP1, by competition with a fluorescently labeled ATP-competitive ligand, as previously described.^18^ In brief, GST-RipK1 (1–375) was purified and was used at a final assay concentration of 10 nM. A fluorescent-labeled ligand (14-(2-{[3-({2-{[4-(cyanomethyl)phenyl]amino}-6-[(5-cyclopropyl-1H-pyrazol-3-yl)amino]-4-pyrimidinyl}amino) propyl]amino}-2-oxoethyl)-16,16,18,18-tetramethyl-6,7,7a,8a,9,10,16,18-octahydrobenzo [2“,3“]indolizino[8“,7“:5ʹ,6ʹ]pyrano[3ʹ,2ʹ:3,4]pyrido[1,2-a]indol-5-ium-2-sulfonate was used at a final assay concentration of 5 nM. Samples were read on an Analyst multimode reader (Molecular Devices, Sunnyvale, CA, USA). Test compound inhibition was expressed as percent (%) inhibition of internal assay controls.

### ADP-Glo kinase assay

Compound potency against RIP1 kinase activity was determined using an ADP-Glo luminescence assay, which measures the conversion of ATP to ADP as previously described.^18^ In brief, the primary reaction consisted of 10 nM GST-RIPK1 (1–375) and 50 *μ*M ATP in 50 mM HEPES pH 7.5, 50 mM NaCl, 30 mM MgCl2, 1 mM DTT, 0.5 mg/ml BSA, and 0.02% CHAPS. Five microliter of enzyme and 5 *μ*l of ATP were added to the plate at twice the final assay concentration and incubated at room temperature for 4 h. The luminescence was measured on a plate reader. Test compound inhibition was expressed as percent inhibition of internal assay controls.

### Kinase selectivity

GSK′963 was tested against 339 kinases using a P33-radiolabeled assay at Reaction Corp Biology (http://www.reactionbiology.com). The compound was tested at a single dose in duplicate at 10 *μ*M. Reactions were carried out at 10 *μ*M ATP. Data are reported as % enzyme activity (relative to DMSO controls). The full data set is shown in the Supplementary Table I.

### IDO enzymatic assay

IDO activity was determined as described by Takahashi *et al.*^25^ using recombinant human IDO purchased from R&D Systems (Minneapolis, MN, USA). The yellow pigment derived from kynurenine was measured at 490 nm using the Spectramax microplate reader (Molecular Devices).

### Cell culture

Mouse fibrosarcoma L929 cells (ATCC# CCL-1) and human monocytic U937 cells (ATCC# CRL-1593.2) were cultured in RPMI media supplemented with 10% heat-inactivated FBS, 100 U/ml penicillin and 100 U/ml streptomycin. BMDM were prepared from C57BL/6 mice by differentiation with 10 ng/ml M-CSF (R&D Systems) for 7 days and cultured in DMEM medium supplemented with 10% heat-inactivated FBS, 100 U/ml penicillin, 100 *μ*g/ml streptomycin and 0.25 *μ*g/ml amphotericin. Primary human neutrophils were isolated from human blood following the standard method comprised of sequential sedimentation in dextran, density centrifugation in Ficoll–Hypaque and lysis of contaminating red blood cells.

### Cell-based assays

Necroptotic cell death was induced in BMDM, L929 and U937 cells with TNF in the presence of caspase inhibitor zVAD-FMK or QVD-Oph (BMDM: 50 ng/ml TNF+50 *μ*M zVAD; L929: 100 ng/ml TNF+50 *μ*M QVD; U937: 100 ng/ml TNF+25 *μ*M QVD). To evaluate the effect of RIP1 inhibitors, cells were pretreated with compound (dose–response) for 30 min. Induced cell death was evaluated 19–21 h later by measuring cellular ATP levels using CellTiter-Glo Luminescent Cell Viability assay (Promega, Fitchburg, WI, USA). To induce necroptosis in neutrophils, freshly isolated human neutrophils were stimulated with TNF (10 ng/ml), QVD-Oph (50 *μ*M) and SMAC mimetic (100 nM). Induced cell death was evaluated as above.

### Caspase 3/7 assay

To induce apoptosis, BMDM pretreated with GSK′963 (100 nM), GSK′962 (100 nM) or Nec-1 (10 *μ*M) for 30 min were stimulated with TNF (50 ng/ml, R&D Systems) and CHX (12 *μ*g/ml, Sigma, St. Louis, MO, USA). Caspase 3/7 activity was measured at 6 h using the Caspase-Glo 3/7 assay (Promega).

### Western blotting

For immunoblot analysis, BMDM pretreated with GSK′963 (100 nM), GSK′962 (100 nM) or Nec-1 (10 *μ*M) for 30 min, were stimulated with 50ng/ml TNF for 5 and 15 min. Lysates prepared in 1× Cell Lysis Buffer (Cell Signaling, Danvers, MA, USA) containing protease and phosphatase inhibitors were separated on 4–12% SDS-PAGE and blotted onto nitrocellulose membrane (Invitrogen, Waltham, MA, USA). Blots were probed for I*κ*B (9242, Cell Signaling), phospho-I*κ*B (2859, Cell Signaling), and tubulin as a loading control.

### Pharmacokinetic *experiments*

Briefly, pharmacokinetic studies were conducted using *n*=3 animals per group for each compound. Animals received either GSK′963 or 7-Cl-O-Nec-1 as an i.p. injection prepared as aqueous solutions in 6% 11-b-hydroxypropyl cyclodextrin and contained 5% DMSO. Blood samples were obtained at various time points following intraperitoneal injection and diluted 1 : 1 with water prior to storage at −80 °C.

Prior to bioanalysis, samples were thawed and each analyte was isolated using a method based on protein precipitation. The resulting supernatant was injected into an LC/MS/MS system optimized for detection of the compound of interest. Data were reported as quantitative drug concentrations as determined by standard calibration curve analysis. Using these optimized conditions, the typical lower limit of quantitation achieved was 1.00 ng/ml for both compounds.

### TNF+zVAD-induced shock model

C57BL/6 mice were pretreated i.p. with Nec-1 (0.2 and 2 mg/kg), GSK′963 (0.2 and 2 mg/kg) or GSK′962 (20 mg/kg) 15 min prior to i.v. injection of TNF (1.25 mg/kg, Cell Sciences, Canton, MA, USA) and zVAD-FMK (16.7 mg/kg, Bachem, King of Prussia, PA, USA). Temperature was monitored over the course of 3 h by rectal probe.

### Statistics

*In vitro* data are shown as mean±S.D. and *in vivo* data are shown as mean±S.E. Dose–response curves and IC_50_ values for biochemical and cell-based assays were generated using GraphPad Prism 5 software (GraphPad Software, Inc., San Diego, CA, USA).
