# Supplementary material for: Characterization of GSK′963: a structurally distinct, potent and selective inhibitor of RIP1 kinase
Source: Cell Death Discov. 2015 Jul 27;1:15009–. doi: 10.1038/cddiscovery.2015.9 (PMC4979471; doi:10.1038/cddiscovery.2015.9)
Supplement: Supplementary Figure 1 [file cddiscovery20159-s1.ppt]

## Slide 1
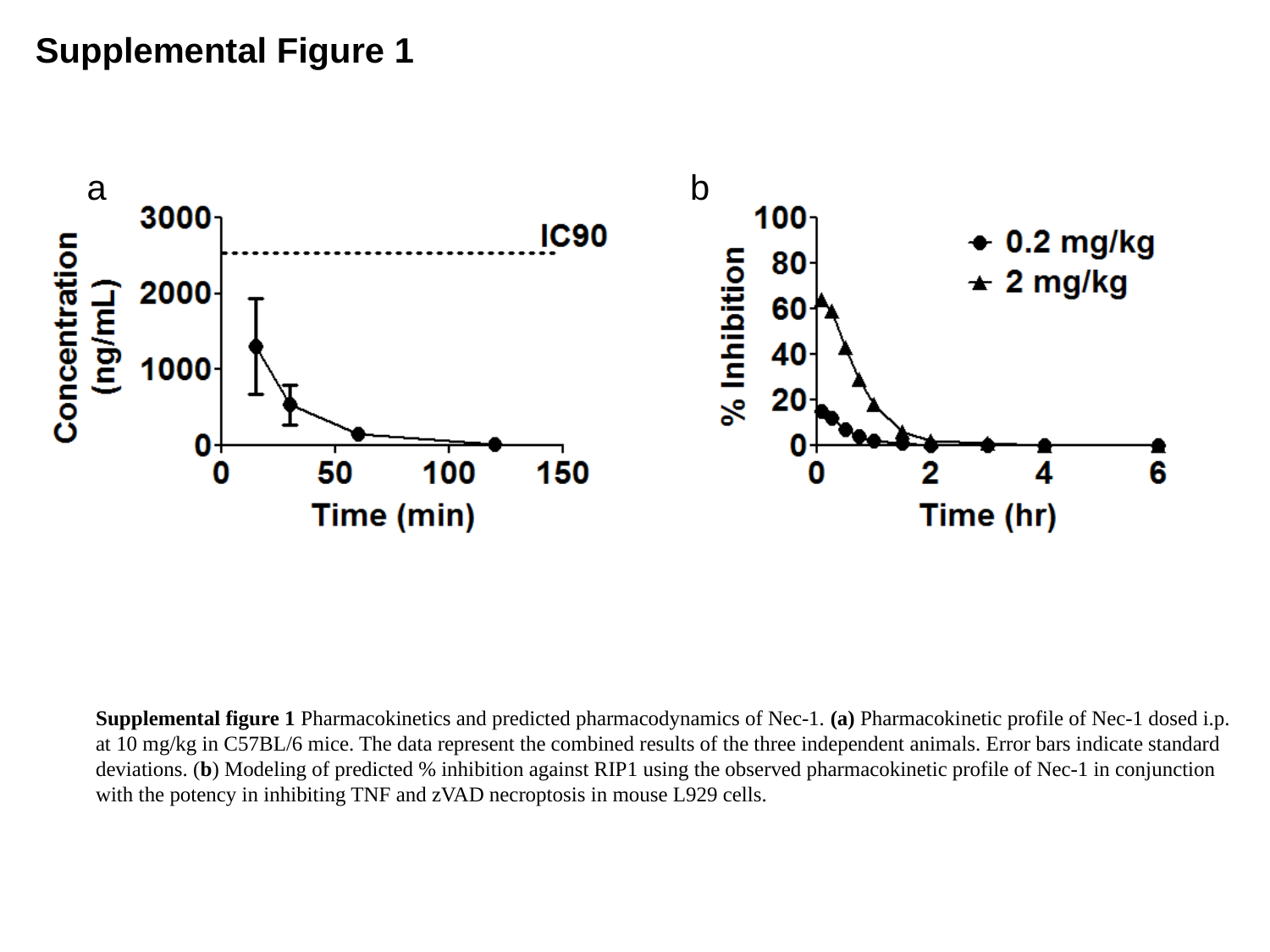

Supplemental Figure 1
a
b
Supplemental figure 1 Pharmacokinetics and predicted pharmacodynamics of Nec-1. (a) Pharmacokinetic profile of Nec-1 dosed i.p.
at 10 mg/kg in C57BL/6 mice. The data represent the combined results of the three independent animals. Error bars indicate standard
deviations. (b) Modeling of predicted % inhibition against RIP1 using the observed pharmacokinetic profile of Nec-1 in conjunction
with the potency in inhibiting TNF and zVAD necroptosis in mouse L929 cells.
